# Drought-induced transposon expression reveals complex drought response mechanisms in *Brassica napus*

**DOI:** 10.3389/fpls.2025.1614169

**Published:** 2025-07-23

**Authors:** Manyi Chen, Dan Luo, Hanzhao Kong, Yan Lv, Chunsheng Li, Yongguo Zhao, Qian Huang, Guangyuan Lu

**Affiliations:** ^1^ Guangdong Provincial Key Laboratory for Green Agricultural Production and Intelligent Equipment, Guangdong University of Petrochemical Technology, Maoming, China; ^2^ Oil Crop Research Institute, Chinese Academy of Agricultural Science, Wuhan, China; ^3^ College of Life Science and Technology, Hubei Engineering University, Xiaogan, China; ^4^ Institute of Crop Science, Zhejiang University, Hangzhou, China; ^5^ School of Biology and Food Engineering, Guangdong University of Petrochemical Technology, Maoming, China

**Keywords:** *Brassica napus*, transposable element (TE), transcript, drought stress, protein-coding TE, non-coding RNA, gene regulation

## Abstract

Transposable elements (TEs) are abundant components of plant genomes, yet their transcriptional activity and potential biological roles remain underexplored, especially under environmental stress conditions. This study investigates the transcriptional dynamics of TEs in *Brassica napus* during drought stress in seed development, aiming to uncover their contributions to stress responses and seed germination. RNA-seq data were analyzed for TE transcriptional activity in wild-type (WT) and *BnaABI5* CRISPR-edited mutant lines of *B. napus*. A comprehensive computational pipeline was used to identify and characterize TE-derived transcripts, including protein-coding and long non-coding RNAs (lncRNAs). Functional annotation was performed for protein-coding TE transcripts located in intergenic regions to predict their involvement in biological processes. Out of 212,800 TEs identified in the *B. napus* genome, 17,547 were transcriptionally active, yielding 15,808 protein-coding transcripts and 1,739 lncRNAs. Among these, 65 protein-coding TE transcripts were identified as transposase genes, while 860 transcripts were predicted to represent novel genes derived from transposon regions, potentially participating in monocarboxylic acid metabolic processes. Specific to drought stress responses during seed germination, 128 protein-coding TE transcripts (including 5 transposases) and 37 lncRNAs were differentially expressed. Notably, the lncRNA transcripts MSTRG.108925.4 and MSTRG.109003.7 were implicated in regulating the PHD finger protein ALFIN-LIKE 1 (*BnA10g0418090*), contributing to drought tolerance mechanisms. This study highlights the functional relevance of TE transcription in the context of drought stress during seed germination, providing novel insights into TE-derived genes and lncRNAs as potential regulators of stress responses. These findings expand the understanding of TE biology in plants and offer valuable resources for future efforts to identify drought-resistant genes in *B. napus*.

## Introduction

The genomes of different plants vary significantly, with a major factor driving these differences being the abundance of TEs. For instance, in *Arabidopsis thaliana*, TEs make up 20% of the genome, while in maize, TEs account for 80% (Schnable et al., 2009; Mirouze and Vitte, 2014). There are two main types of TEs: Class I TEs, also known as RNA TEs or retrotransposons, which transpose via reverse transcription of RNA, and Class II TEs, also known as DNA TEs, which transpose through the action of transposases encoded by the TE itself. Retrotransposons include two major categories: long terminal repeat retrotransposons (LTR-RTs) and non-LTR retrotransposons, both of which are highly abundant in plants. For example, LTRs comprise 70% of the maize genome. TE sequences were labeled as “junk DNA” (Gorbunova et al., 2021) due to the limited understanding of their functional roles.

In recent years, there has been a growing understanding of the functions of TE sequences in plant genomes. TE insertions in exon or intron regions of genes are common and can lead to the production of new transcripts (Luehrsen and Walbot, 1990) or alter gene expression levels, either enhancing or reducing them (West et al., 2014; Kim et al., 2015). When these TEs insert not within genes but in upstream regions, they can influence gene expression by affecting regulatory elements, potentially impacting transcriptome evolution and functional outputs (Faulkner et al., 2009). Such TEs can not only serve as alternative promoter sequences but also activate the transcription of new transcripts (Morgan et al., 1999). Conversely, some TEs may disrupt existing promoter structures, resulting in reduced transcription levels (Ramakrishnan et al., 2022).

Recent studies have revealed that, beyond influencing gene transcription and expression, TEs can also produce functional transcripts (Jurka et al., 2007). The accumulation of sequence variations within TEs, along with natural selection, has caused some TEs to lose their transpositional ability. These TEs may have evolved into non-coding RNAs that potentially carry important functions beneficial for host survival (Naville et al., 2016). Long non-coding RNAs (lncRNAs), which are non-coding RNAs longer than 200 nt, are key regulatory factors in gene expression (Mach, 2017; Qin et al., 2017). Given that plants are sessile organisms, mechanisms for stress response and adaptation are critical areas of research. LncRNAs play significant regulatory roles in plant stress responses (Traubenik et al., 2024). In plants like rice, Arabidopsis, and maize, a large number of lncRNAs transcribed from TE sequences have been identified. For instance, compared to wild-type (WT), Arabidopsis seedlings lacking the TE-*lincRNA11195* show increased resistance to abscisic acid (ABA) treatment, suggesting that this lincRNA is involved in abiotic stress responses (Wang et al., 2017).

The regulation of abiotic stress responses in rapeseed is closely associated with non-coding RNAs like lncRNAs (Tan et al., 2020; Waseem et al., 2022). However, systematic studies specifically focusing on TE expression patterns and their contributions to stress responses and lncRNA formation are still lacking. Abscisic acid-insensitive 5 (ABI5) is a key transcription factor (TF) that plays a central role in the regulation of plant responses to various environmental stresses, particularly in drought tolerance and seed dormancy (Skubacz et al., 2016). It is a member of the basic leucine zipper (bZIP) family of TFs, which are known to regulate gene expression through binding to specific DNA motifs in the promoter regions of target genes (Collin et al., 2021). Studies have shown that ABI5 regulates stress tolerance, seed maturation, and germination by mediating the effects of abscisic acid (ABA) (Lopez-Molina et al., 2001; Huang et al., 2008; Sharma et al., 2011; Skubacz et al., 2016). In this study, we performed a comprehensive analysis of TE expression patterns based on transcriptome data from WT and mutant rapeseed under drought stress and control conditions. Additionally, by integrating genotypic information, we explored changes in the regulatory networks of non-coding RNAs derived from TEs. We identified a series of TE-derived transcripts (TE transcripts) responsive to drought stress with potential biological functions, providing a foundation for further understanding the mechanisms underlying seed germination under drought conditions in rapeseed.

## Materials and methods

### Data source

The transcriptomic sequencing data of WT (ZS6, *Brassica napus* variety “Zhongshuang 6”) and mutant transgenic rapeseeds under PEG, exogenous ABA treatment was obtained from a previous study in our lab (NCBI, BioProject accession number PRJNA1227215). Detailed information about the WT and mutant lines is described in detail in a previously published study (Luo et al., 2025). The specific treatments are as follows: WT and mutant lines were placed in dual-layer filter paper culture dishes (10 cm × 10 cm) containing water, 10% PEG, and 2 μM ABA solution, and incubated in a 25°C light-controlled incubator with three biological replicates. Samples were collected and sequenced at 0 h, 24 h, 48 h, and 72 h post-treatment.

### Identification of TEs and quantification of TEs and transcripts

The reference genome files and GFF file for *B. napus* were obtained from the Brassicaceae Database (http://brassicadb.cn/#/) (Chen et al., 2022), using version Brana_ZS_V2.0. Transposable element (TE) annotation of the genome was performed using the Extensive *de novo* TE Annotator (EDTA, v1.9.6) (Ou et al., 2019).

For transcriptome sequencing, raw data were processed with fastp (Chen, 2023) to remove adapter sequences and low-quality reads, resulting in clean data. The clean reads were aligned to the reference genome using Bowtie2 (Langmead and Salzberg, 2012), followed by BAM file sorting with Samtools (Danecek et al., 2021). Transcript assembly was performed with StringTie (Pertea et al., 2015), generating a GTF file for each BAM file. These GTF files were then merged using StringTie. The overlap regions between transcripts and TEs were extracted using Bedtools (Danecek et al., 2021) (parameters: -loj -wa -f 0.9). Transcripts overlapping more than 90% with TEs were considered TE-derived transcripts. The fastq files were re-mapped to the reference genome using STAR (Dobin et al., 2013), with both the reference genome GTF and the TE-derived transcript GTF provided separately as inputs for the –sjdbGTFfile option and –quantMode TranscriptomeSAM specified. The resulting transcriptome-based BAM files were used as input for RSEM to quantify the TE transcripts, while gene expression was quantified using featureCounts (Liao et al., 2014). The expression levels of transposable elements were quantified using TEspeX (https://github.com/fansalon/TEspeX) (Ansaloni et al., 2022). For the expression levels of TEs, TE transcripts, and genes, HTSeq (Putri et al., 2022) was used to convert read counts into FPKM (Fragments Per Kilobase of exon model per Million mapped fragments).

### Differential expression analysis

Genes, TEs, or TE transcripts with an average FPKM ≥ 1 across all samples were considered expressed in each abiotic stress experiment. Differential expression analysis for the comparisons PEG treatment vs. H_2_O and ABA treatment vs. H_2_O at three different time points (24 h, 48 h, and 72 h) was performed using DESeq2 (Love et al., 2014). Upregulated genes were defined as having a log2 fold change > 1 and adjusted *P* ≤ 0.05, while downregulated genes were defined as having a log_2_|fold change| < -1 and adjusted *P* ≤ 0.05. The differential expression results for genes and TE transcripts across different groups were visualized using the UpSet R package (Conway et al., 2017).

### PCA and correlation analysis of TE expression levels

The expression levels of TEs were log-transformed as log2(FPKM + 1), and principal component analysis (PCA) was performed using the prcomp function in R. The first two principal components were visualized with ggplot2 (Villanueva and Chen, 2019). The pheatmap package (Gu et al., 2016) was used to generate clustered heatmaps showing both sample correlations and TE expression levels, with sample correlations calculated using the cor function in R (the “method” parameter was set to “pearson”).

### Prediction of protein-coding TE transcripts and lnc TE transcripts

TE transcripts longer than 200 bp, with more than two exons and expression levels greater than 5 reads, and classified by gffcompare (Pertea and Pertea, 2020) with class codes “u,” “x,” and “i,” were selected for coding potential prediction. Three tools (PLEK (Li et al., 2014), CPAT (Wang et al., 2013), and CPC2 (Kang et al., 2017)) were used to assess the coding potential of these transcripts. Transcripts predicted as non-coding by both PLEK and CPC2, and with a predicted coding probability (Coding_prob) < 0.364 according to CPAT, were classified as lnc TE transcripts. Other transcripts that were predicted to have protein-coding potential by these tools were classified as protein-coding TE transcripts.

### Prediction of transposase in TE transcript

First, we downloaded the Pfam-A.hmm file from InterProScan (https://www.ebi.ac.uk/interpro/). Using HMMER (Johnson et al., 2010), we searched for Pfam-A.hmm within TE transcripts to identify the Pfam domains present in each TE transcript. We searched Pfam domains associated with transposases (**Supplementary Table S1**). TE transcripts containing these transposase-related Pfam domains were classified as transposase.

### Quantitative real time-PCR determination

Total RNA was extracted from different leaf samples of *B. napus* using the Plant RNA Purification Reagent (Invitrogen, Carlsbad, CA, USA). Genomic DNA contamination was removed with DNase I treatment (Invitrogen), and first-strand cDNA was synthesized from 2 μg of total RNA using the Omniscript RT Kit (Qiagen, Valencia, CA, USA). Gene-specific primers (**Supplementary Table S2**) were designed using Primer Premier 5.0 (Lalitha, 2000) and verified for specificity through BLAST searches against the B. napus genome. Quantitative real-time PCR (qRT-PCR) was performed using the LightCycler 480 system (Roche), with amplification reactions conducted according to the instructions of the LightCycler 480 SYBR Green I Master Kit (Roche). The Actin7_141 gene was used as an internal reference (Lou et al., 2024). Each sample was analyzed in three biological replicates. Relative gene expression levels were calculated using the 2^–ΔΔCt^ method (Holzapfel and Wickert, 2007), based on the average threshold cycle (Ct) values for each sample.

## Results

### Expression of TEs in the *Brassica napus* genome

**Figure 1:**
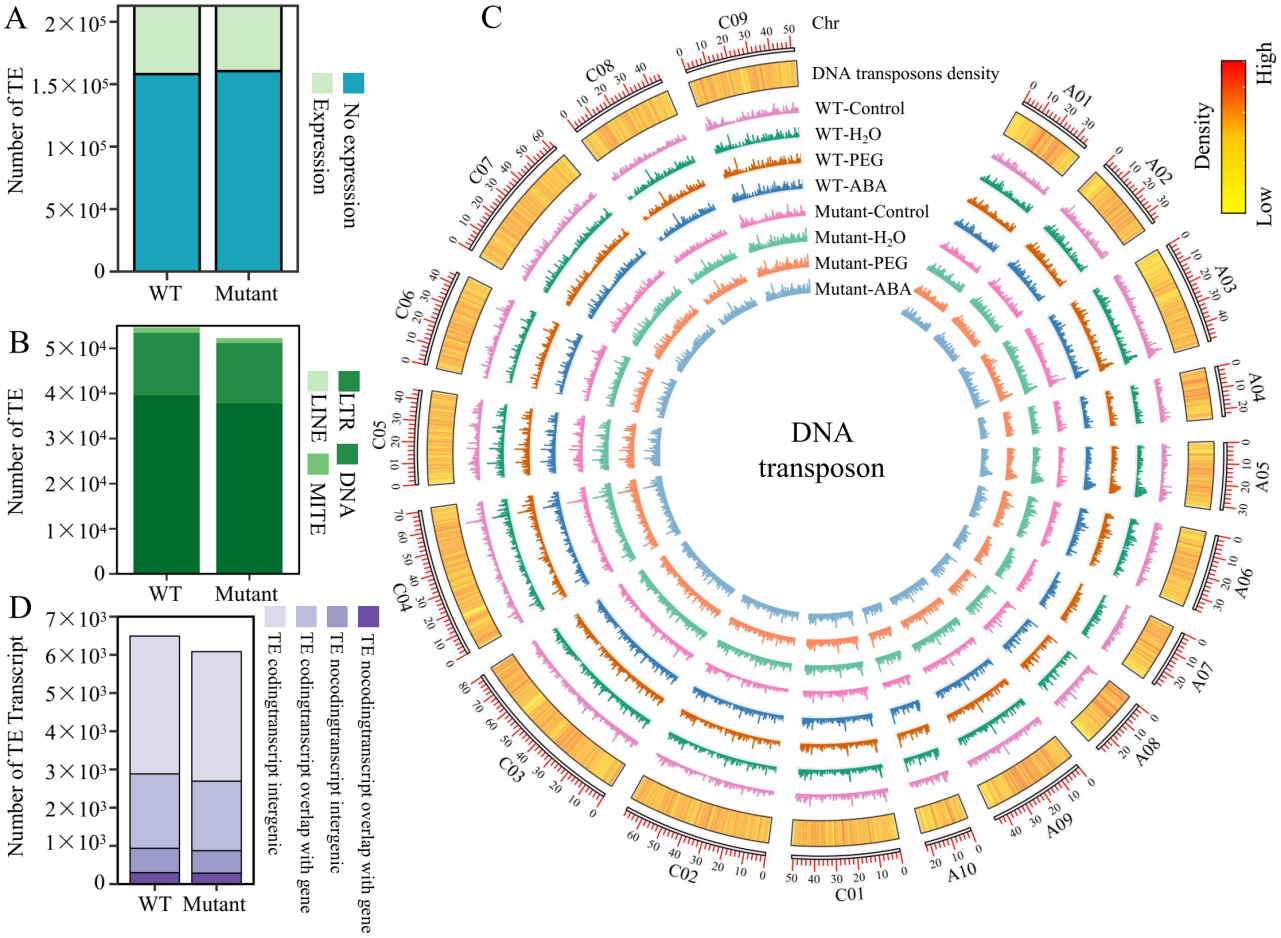
Widespread expression and classification of TEs in *B. napus*. **(A)** Stacked bar chart showing the number of expressed and non-expressed TEs in WT and mutant lines (TEs with an average FPKM ≥ 1 across all samples were considered expressed). **(B)** Number of expressed TEs of different types detected in WT and mutant lines. **(C)** Density distribution of DNA transposons across chromosomes and their expression patterns in various samples. The circos plot from outer to inner rings shows chromosomes, DNA transposons density within 50 kb windows, and expression levels under different treatments across different samples within 50 kb windows. Mutant represents mutant lines. Control represents treatment after 0 h. H_2_O, PEG, and ABA represent different samples after H_2_O, PEG, and ABA treatments, respectively. **(D)** Number of TE transcripts relative to their genomic positions with respect to genes in WT and mutant lines.

In this study, we identified 212,800 TEs in the *B. napus* genome (Brana_ZS_V2.0), including 135,188 DNA transposons, 72,892 LTRs, 451 LINEs (Long Interspersed Nuclear Elements), and 4,269 MITEs (Miniature Inverted-repeat Transposable Elements). Our previous research revealed that BnaABI5 regulates ABA to mediate seed germination under drought conditions. Given that transcriptional activity in TE regions is associated with various biological processes, we quantified TE expression levels using RNA-seq data from 60 WT and mutant lines under different treatments. In the WT and mutant lines, 54,734 (25.72%) and 52,349 (24.6%) TEs were expressed, respectively (**Figure 1A**). Among them, DNA transposons had the highest number of expressed TEs (39,529 in the WT and 37,758 in the mutant lines), while LINEs had the fewest (146 in the WT and 162 in the mutant lines). There were 13,897 and 13,414 expressed LTRs, while there were 1,162 and 1,051 MITEs in the WT and mutant lines, respectively (**Figure 1B**). The chromosomal expression patterns of most TEs were similar across samples, but some genomic regions showed significant changes in TE expression between the control (treatment after 0h) and all treated groups. For example, the transcriptional abundance of DNA transposons was higher in the treated groups near the 22 Mb region (21406762- 22216053) on chromosome A10, the 18 Mb region (17630418- 18028290) on chromosome C08, and the 10 Mb region (10099783- 10969962) on chromosome C09 compared to the control (**Figure 1C**). The average transcript abundance of other transposons in all samples was LTR>MITE>LINE (**Supplementary Figure S1**). Changes in epigenetic modifications due to abiotic stress may contribute to these differences in TE expression (Liang et al., 2021), and these altered TE transcripts may play a role in the seed germination of *B. napus* under drought stress.

Additionally, this study predicted the protein-coding potential of transcripts derived from TE regions. A total of 5,555 and 5,192 protein-coding TE transcripts were identified in WT and the mutant lines, respectively. For TE transcripts lacking protein-coding potential, we classified them as lnc RNAs, with 936 identified in WT and 874 in the mutant lines. We found that protein-coding TE transcripts were primarily located in intergenic regions. In WT and the mutant lines, 3,607 and 3,390 TE transcripts with protein-coding potential were located in intergenic regions, respectively (**Figure 1D**). Based on the annotated protein domains of these TE transcripts with protein-coding potential in intergenic regions, we identified 884 TE transcripts with Pfam protein domain annotations (**Supplementary Table S3**), among which only a minority (65) were associated with transposases, likely due to the inherent nature of TEs as mobile genetic elements. Additionally, we identified 4754 TE transcripts without annotated transposase-related domains, suggesting the potential emergence of novel functions in these TE transcripts.

### The expression characteristics of TEs

**Figure 2:**
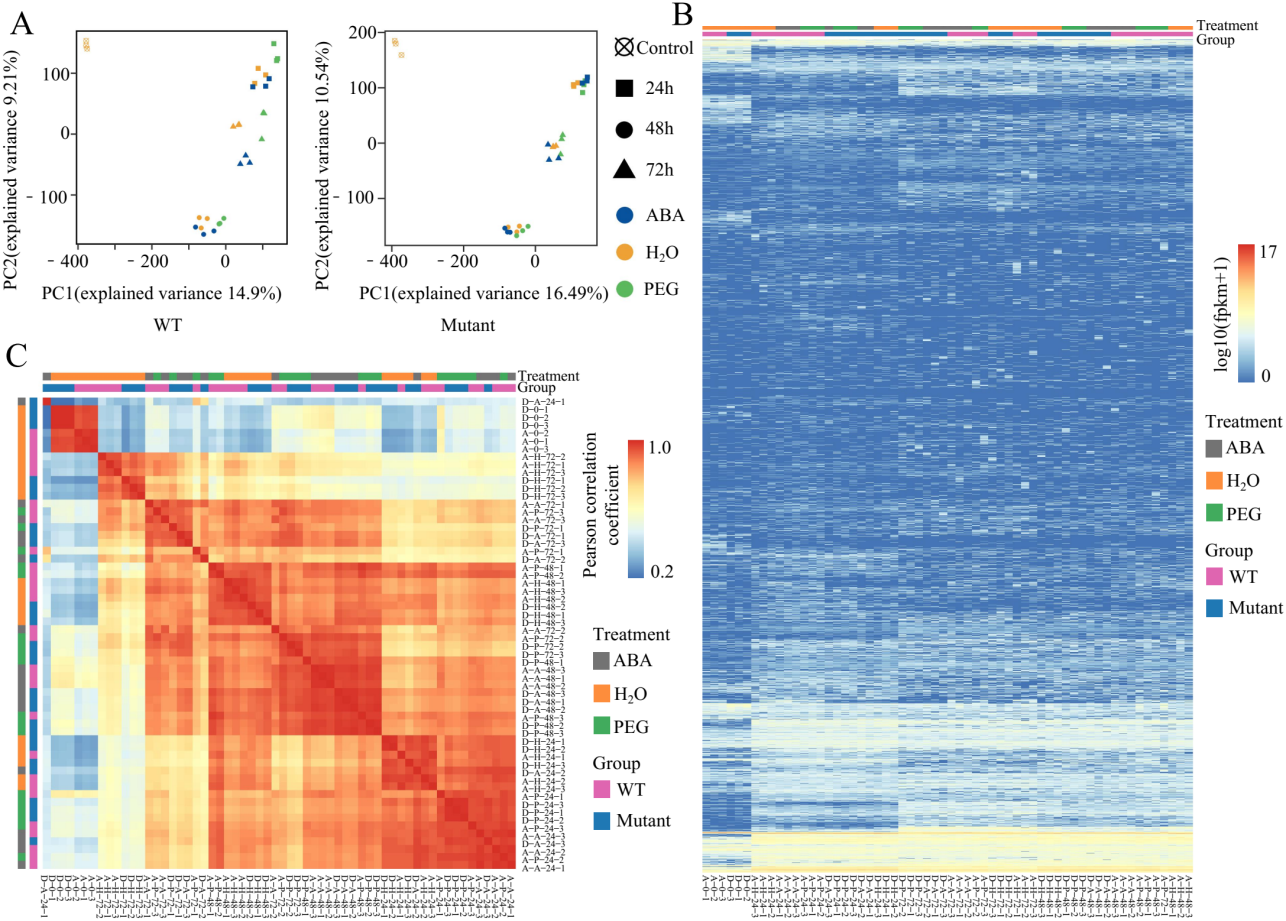
Transcriptional characteristics of TEs in WT and mutant lines under different treatments and time points. **(A)** PCA analysis of TE expression profiles in WT and mutant lines under different treatments. **(B)** Heatmap showing the hierarchical clustering of expression levels for 53,631 TEs across different samples. Mutant represents mutant lines. **(C)** Heatmap of the PCC based on the expression levels of 53,631 TEs across different samples. Mutant represents mutant lines. The first letter A or D in the sample name represents WT and Mutant, respectively, the second letter (H, A, P) represents different treatments (H_2_O, ABA, PEG, and none represents untreated), the first number represents the treatment time (0h, 24h, 48h, and 72h), the second number represents the 3 replicates.

To explore the expression characteristics of TEs under different treatment conditions and time gradients, we systematically analyzed the TE expression patterns in the WT and mutant lines across various treatments and time points. PCA analysis of the TE expression profiles revealed similarities and differences among the samples under different treatments and time points. The PCA analysis showed that at the same time point, even under different stress treatments, the TE expression profiles of the samples were still highly similar. However, under the same stress treatment, the differences between samples at various time points were more significant, indicating that the influence of time factors on TE expression changes is greater than that of stress treatments (**Figure 2A**). This finding suggests that during seed germination, the expression levels of TEs are mainly regulated by developmental stages, while stress treatments have a relatively minor impact, a trend that is more pronounced in the mutant lines.

Further clustering analysis of TE expression revealed differences in the response of TEs to abiotic stress and developmental stages (**Figure 2B**). Most TEs exhibited low transcription levels. However, in both the WT and mutant lines, some TEs showed high expression only after stress treatments, while maintaining low expression in control samples (0 h after treatment). Clustering analysis based on TE transcription levels indicated that samples under different stress treatments exhibited similar expression patterns, and at the same time point, the samples from both genotypes also showed similar expression characteristics. This further confirms that during rapeseed seed germination, TE transcription patterns are more consistent across the same time point than across different stress treatments. To further confirm the features of TE expression patterns, we calculated and clustered the Pearson Correlation Coefficient (PCC) of TE expression levels (FPKM values) across multiple samples (**Figure 2C**). The results verified that the changes in TE transcription are primarily influenced by time factors, followed by stress treatment. These findings provide new insights into the biological functions of TE transcription during plant development and stress response, offering a foundation for further research.

### Expression patterns of protein-coding TE transcripts

**Figure 3:**
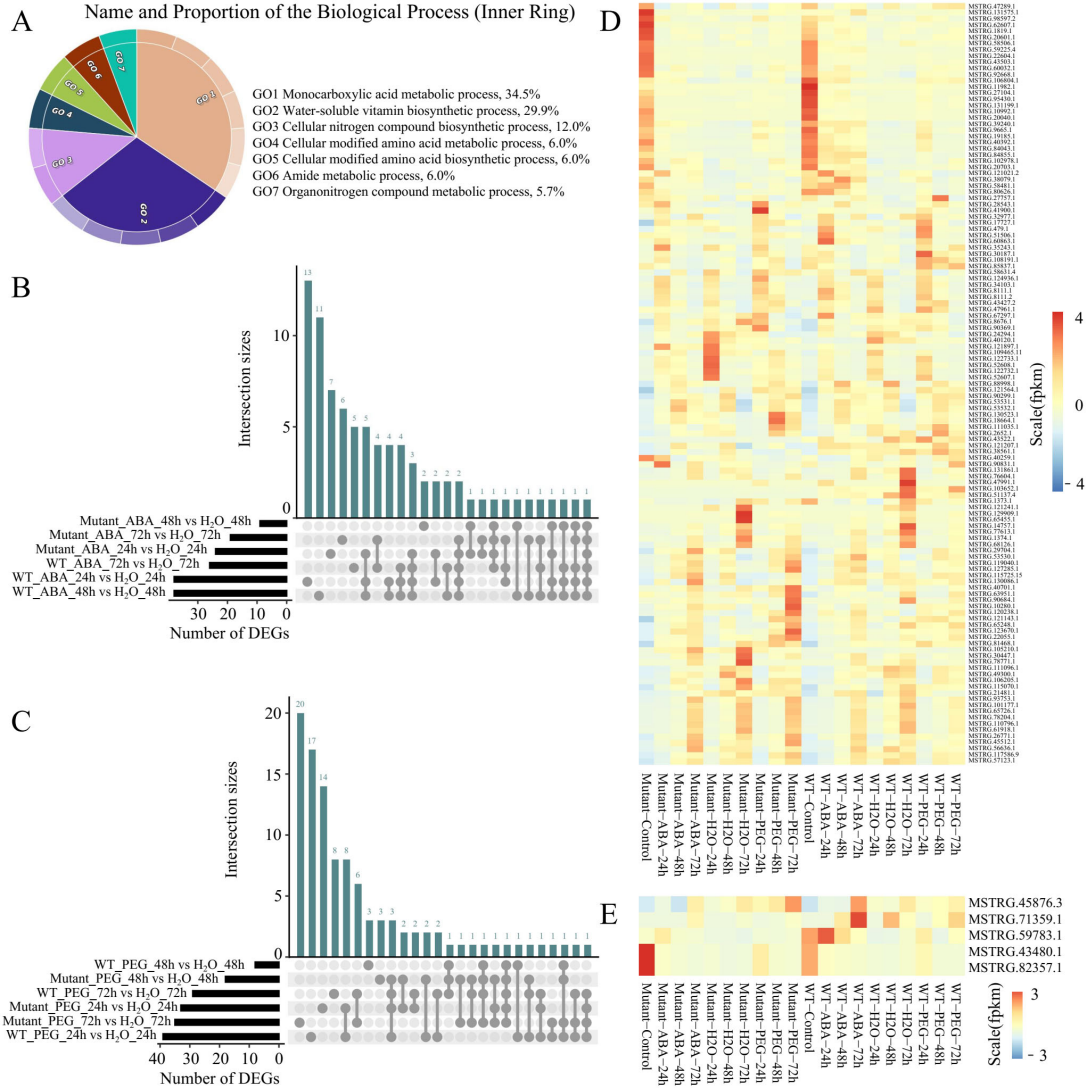
Expression characteristics of protein-coding TE transcripts. **(A)** GO enrichment analysis of protein-coding TE transcripts. **(B)** Upset plot of differentially expressed protein-coding TE transcripts in WT and mutant lines under ABA treatment at different time points. **(C)** Upset plot of differentially expressed protein-coding TE transcripts in WT and mutant lines under PEG treatment at different time points. **(D)** Expression levels of 128 differentially expressed protein-coding TE transcripts across samples. **(E)** Expression levels of five transposase-encoding differentially expressed protein-coding TE transcripts across samples.

TE transcripts were classified into 15,808 protein-coding TE transcripts and 1,739 non-coding TE transcripts. Functional enrichment analysis revealed that 4,832 protein-coding transcripts located in intergenic regions are primarily enriched in biological processes such as monocarboxylic acid metabolic process (34.5%), water-soluble vitamin biosynthetic process (29.9%), cellular nitrogen compound biosynthetic process (12%), and cellular modified amino acid metabolic process (6.0%) (**Figure 3A**). These findings indicate that TE regions transcribe transcripts not only encoding transposase-related enzymes but also produce transcripts associated with proteins involved in other biological functions. Furthermore, these protein-coding TE transcripts play an important role in the synthesis and metabolism of various compounds. This broadens our understanding of TE transcription biology and offers clues for uncovering TEs with novel biological functions.

This study comprehensively revealed the expression patterns of protein-coding TE transcripts under various stress treatments and dynamic time courses. Compared to water treatment, WT plants exhibited 38, 38, and 25 differentially expressed protein-coding TE transcripts after 24, 48, and 72 hours of ABA treatment, respectively. In contrast, the mutant lines showed 23, 8, and 18 differentially expressed protein-coding TE transcripts at the corresponding time points (**Figure 3B**, **Supplementary Table S4**). Combined with our findings that mutant lines display higher seed germination rates under drought treatment, this suggests that the absence of *BnaABI5* promotes seed germination under stress conditions, potentially by disrupting the regulatory networks between genes and between genes and non-coding RNAs (ncRNAs), which in WT plants may inhibit germination as a survival strategy during stress. This further indicates that these TE transcripts may be embedded within these intricate interaction networks and are regulated by the *BnaABI5* mutation. Additionally, under PEG treatment, 39, 7, and 28 differentially expressed protein-coding TE transcripts were identified in WT plants at 24, 48, and 72 hours, respectively, compared to water treatment, while the mutant lines exhibited 32, 17, and 34 differentially expressed protein-coding TE transcripts at the same time points (**Figure 3C**). Notably, there was minimal overlap in the differentially expressed protein-coding TE transcripts identified under drought stress conditions between the two genotypes (32 transcripts), with the majority of differentially expressed protein-coding TE transcripts (65 transcripts) being specific to one genotype under a particular time point after the different stress conditions. This further demonstrates that the *BnaABI5* CRISPR editing altered the drought response patterns in *B. napus*.

Among the 128 differentially expressed protein-coding TE transcripts (**Figure 3D**), five transcripts (MSTRG.43480.1, MSTRG.45876.3, MSTRG.59783.1, MSTRG.71359.1, and MSTRG.82357.1) encode proteins with transposase-related functional domains. Additionally, 27 TE transcripts, including MSTRG.47289.1, MSTRG.131575.1, and MSTRG.98597.2, exhibited high expression levels at 0 hours after ABA and PEG treatments but showed relatively lower expression at 24, 48, and 72 hours of treatment. In contrast, nine TE transcripts, such as MSTRG.10280.1, MSTRG.40701.1, and MSTRG.123670.1, displayed higher expression levels in the mutant lines at 24 hours of PEG treatment compared to other treatment groups, while their expression was significantly lower in WT plants after 72 hours of PEG treatment (**Figure 3E**). To further validate the differential expression results, we randomly selected six TE transcripts (MSTRG.40701.1, MSTRG.43480.1, MSTRG.65455.1, MSTRG.90684.1, MSTRG.129909.1, and MSTRG.10280.1) for qRT-PCR analysis (**Supplementary Figure S2**). The qRT-PCR results showed that the relative expression levels and expression patterns of these TE transcripts across different samples were largely consistent with the RNA-seq data, supporting the reliability of our transcript quantification. These results indicates that the proteins translated from these nine differentially expressed protein-coding TE transcripts are primarily involved in drought response at 72 hours.

### Lnc TE transcript identification and differential expression analysis

**Figure 4:**
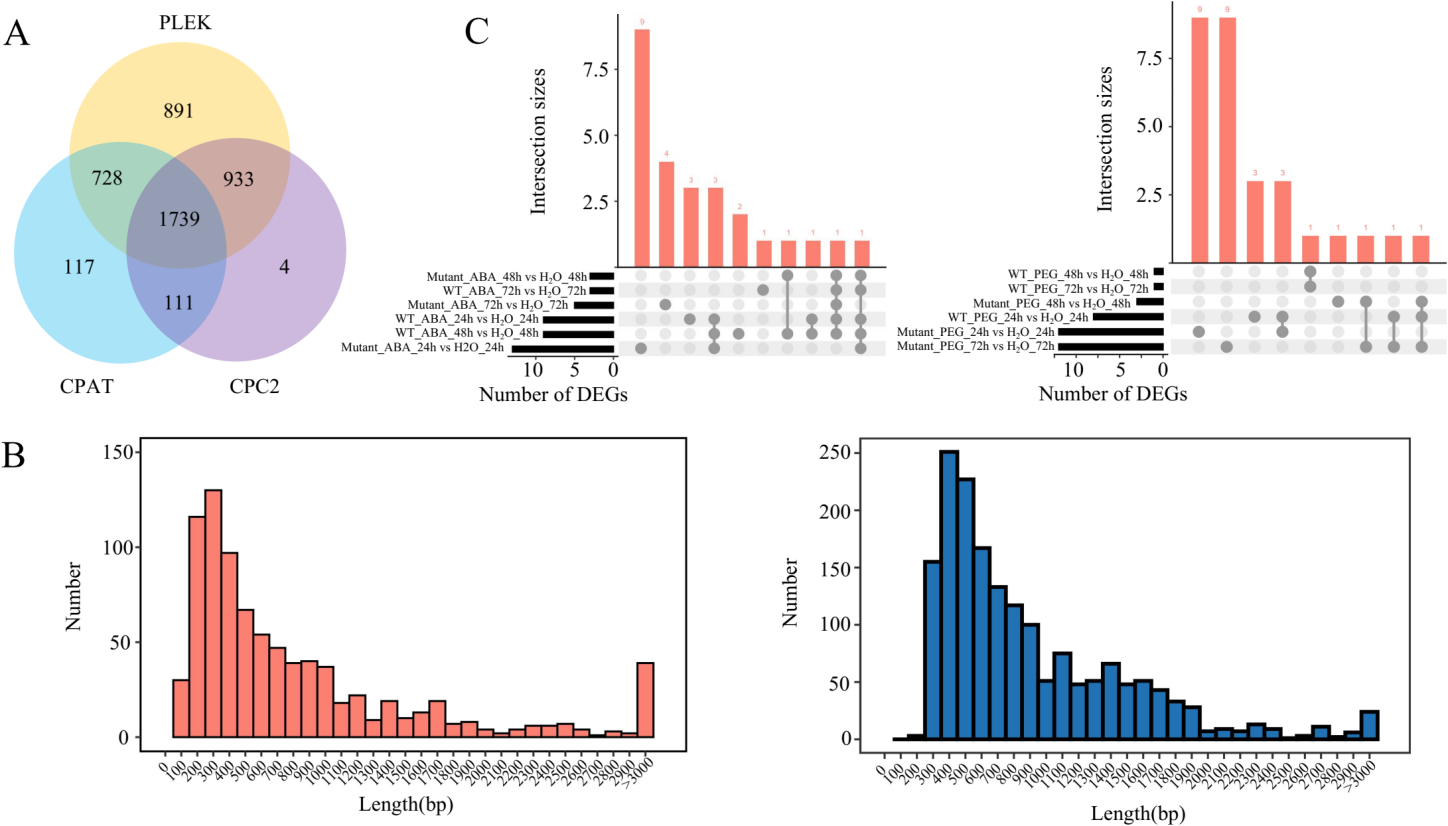
Identification and characteristics of lnc TE transcripts. **(A)** Venn diagram showing lnc TE transcripts identified by different software tools (PLEK, CPAT and CPC2). **(B)** Frequency distribution of the transcript lengths for protein-coding (orange) and lnc (blue) TE transcripts. **(C)** Upset plots of differentially expressed lnc TE transcripts under ABA treatment (left) and PEG treatment (right) at different time points compared to water treatment.

In addition to encoding proteins, TEs can regulate gene expression by transcribing lncRNA. In this study, 1,739 lnc TE transcripts were identified among 17,547 TE transcripts (**Figure 4A**). The length distribution of lnc TE transcripts is similar to that of protein-coding TE transcripts, with the majority falling within the 100–700 bp range. Among the protein-coding TE transcripts, 37 were longer than 3,000 bp, while 22 lnc TE transcripts exceeded this length (**Figure 4B**). These results suggest that lnc TE transcripts exhibit diverse length distributions and may share certain structural characteristics with protein-coding TE transcripts. Compared to water treatment, 9, 9, and 3 differentially expressed lnc TE transcripts were identified in WT plants after 24 h, 48 h, and 72 h of ABA treatment, respectively. 13, 3, and 5 differentially expressed lnc TE transcripts were identified in mutant lines under the same treatment and time points (**Figure 4C**). Similarly, compared to water treatment, WT plants exhibited 8, 1, and 1 differentially expressed lnc TE transcripts after 24 h, 48 h, and 72 h of PEG treatment, respectively. Meanwhile, 12, 3, and 12 differentially expressed lnc TE transcripts were identified in mutant lines under the same conditions. These findings suggest that lnc TE transcripts play distinct roles in response to abiotic stresses and that the mutation of *BnaABI5* alters the expression patterns of these transcripts under stress conditions. These TE-derived lnc transcripts provide a new resource for identifying functional non-coding RNAs.

### Regulations of protein-coding genes by lnc TEs

**Figure 5:**
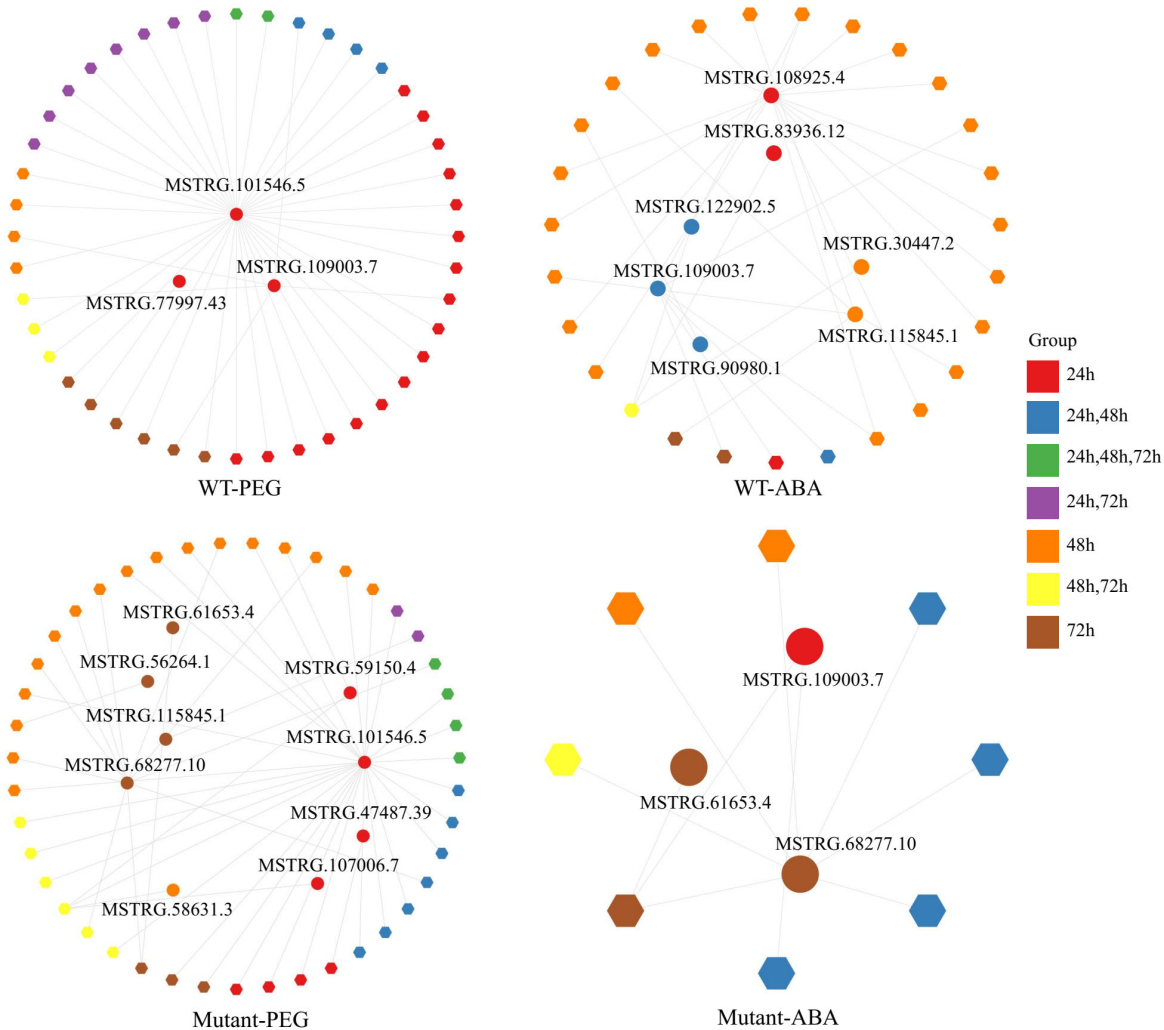
Co-expression networks of lnc TE transcripts under different treatments in WT and mutant lines.

Given that lncRNAs regulate gene expression, we analyzed the regulation of protein-coding genes with differential expression under various treatments by lnc TE transcripts through co-expression analysis. Four co-expression networks were constructed for different genotypes and treatments (**Figure 5**, **Supplementary Table S5**). There were 16 differentially expressed TE transcripts (**Supplementary Table S6**). Under PEG treatment, the co-expression network in WT contained 3 lnc TE transcripts and 44 DEGs, while that in the mutant lines included 9 lnc TE transcripts and 43 DEGs. Notably, the lnc TE transcript MSTRG.101546.5 was co-expressed with multiple DEGs in both WT (38 DEGs) and mutant lines (28 DEGs), suggesting that this transcript may act as a key regulatory factor involved in gene expression under PEG stress, with conserved functions across different genotypes. Under ABA treatment, the co-expression network in WT included 7 lnc TE transcripts and 27 DEGs. Among them, MSTRG.108925.4 was co-expressed with 16 DEGs, while MSTRG.109003.7 was co-expressed with 6 DEGs. Further analysis revealed that both lnc transcripts regulated *BnA10g0418090.1* (encoding PHD finger protein ALFIN-LIKE 1) and *BnC07g0801050.1* (encoding maltose excess protein 1, chloroplastic-like), indicating that lnc TEs may participate in ABA signaling or related biological processes by regulating these key genes. In the mutant lines under ABA treatment, the co-expression network contained 3 lnc TE transcripts and 8 DEGs. Among these, MSTRG.109003.7, MSTRG.61653.4, and MSTRG.68277.10 were all found to regulate *BnC06g0773950.1* (encoding metal tolerance protein 9), suggesting that these lnc TE transcripts may be associated with metal ion homeostasis or resistance mechanisms.

## Discussion

TEs are DNA sequences capable of autonomously replicating or moving within the genome of an organism. Previous studies have primarily focused on the effects of TE insertions and deletions on plant traits (Li et al., 2024; Liu et al., 2024). However, this study shifts the focus to the transcription of TEs. In the transcriptomes of the rapeseed variety ZS6 and its mutant lines under drought stress during seed germination, 6499 and 6104 TE transcripts were identified, respectively. This study reveals the extensive expression of TEs, with not only TEs from various genomic regions being expressed, but also the detection of expression from different types of TEs (such as DNA, LTR, LINE, MITE, etc.). Whether the transcripts of these TEs encode proteins determines the biological functions they can perform. Therefore, this study predicts the coding potential of these transcripts and identifies 15,808 TE transcripts with protein-coding capability and 1,739 non-coding TE transcripts.

TEs can transpose through two mechanisms: copy-and-paste and cut-and-paste, processes that are mediated by enzymes such as integrase or transposase (Goerner-Potvin and Bourque, 2018; Gorbunova et al., 2021). Therefore, TE transcripts with protein-coding potential typically encode enzymes involved in the transposition process. Among the TE transcripts identified in this study, 65 were classified as transposases. Analysis of the functional domains of the proteins encoded by these TE transcripts revealed that 4,767 of them may participate in biological processes beyond transposition. This could be due to the accumulation of mutations and natural selection over evolutionary time, leading to the loss of the original transposase function and the evolution of new roles (Fan et al., 2023). Since genome annotation in plants and animals typically focuses on sequences after masking repeat regions, these newly functionalized TEs are often overlooked in RNA-seq data analysis pipelines. However, the TE transcript quantification software TEspeX used in this study provides a more accurate quantification of transcripts from TE regions (Ansaloni et al., 2022). Consequently, this study identified 4,832 protein-coding TE transcripts, which were enriched in 17 GO terms related to the synthesis and metabolism of compounds, such as monocarboxylic acid metabolic process (34.5%) and water-soluble vitamin biosynthetic process (29.9%). These findings provide valuable resources for further investigation into the newly acquired functions of TEs, especially 125 differentially expressed non-transposase protein-coding TE transcripts under drought stress.

In addition to evolving new functional proteins, TE regions also transcribe non-coding RNAs with biological functions. Several studies have identified non-coding RNAs transcribed from transposon regions that participate in the regulation of plant growth (Li et al., 2022; Zheng et al., 2024). In this study, based on accurate quantification of TE region transcripts, we identified 15 and 31 lnc TE transcripts in WT and mutant lines, respectively, that respond to drought stress. These findings provide valuable resources for exploring functional lncRNAs and enrich our understanding of TEs. Previous research has shown that the Alfin-like transcription factor family, including *AL6* and *AL7*, is preferentially expressed in seeds of Arabidopsis, where the AL PHD-PRC1 complex activates *H3K4me3* transcription and shifts to *H3K27me3* during seed germination (Molitor et al., 2014). Similarly, PHD-finger proteins have been implicated in ABA-mediated stress responses in *Glycine max* (Wang et al., 2015). In line with these studies, our research identified a differential expression of the PHD finger protein *ALFIN-LIKE 1* (*BnA10g0418090*) under ABA treatment. Notably, we also found that the lnc TE transcripts MSTRG.108925.4 and MSTRG.109003.7, which were upregulated and downregulated under drought stress, respectively, co-expressed with *BnA10g0418090* in a shared network. This suggests that these two lnc RNAs may have potential regulatory roles during seed germination under drought stress. It is important to note that while these co-expression patterns point to possible regulatory relationships between TE-derived lncRNAs and transcription factors, the regulatory mechanisms remain to be elucidated in future experimental work. In conclusion, this study quantitatively analyzed the transcription levels and expression patterns of TE regions across the entire genome, providing insights into both protein-coding and lnc TE transcripts. These findings offer a foundation for further exploration of the functional roles of TE regions and contribute to the advancement of functional genomics research.

## Data Availability

The datasets presented in this study can be found in online repositories. The names of the repository/repositories and accession number(s) can be found in the article/**Supplementary Material**.

## Supplementary material

Supplementary Figure 1 Density distribution of LINE, LTR and MITE transposons across chromosomes and their expression patterns in various samples. The circos plot from outer to inner rings shows chromosomes, transposon density within 50 kb windows, and expression levels under different treatments across different samples within 50 kb windows.

Supplementary Figure 2 The bar chart shows the relative expression levels quantified by qRT-PCR of six TE transcripts. The error line represents the standard deviation of three repetitions.
