# Supplementary figures and images for: Drought-induced transposon expression reveals complex drought response mechanisms in Brassica napus
Source: Front Plant Sci. 2025 Jul 23;16:1614169. doi: 10.3389/fpls.2025.1614169 (PMC12325175; doi:10.3389/fpls.2025.1614169)

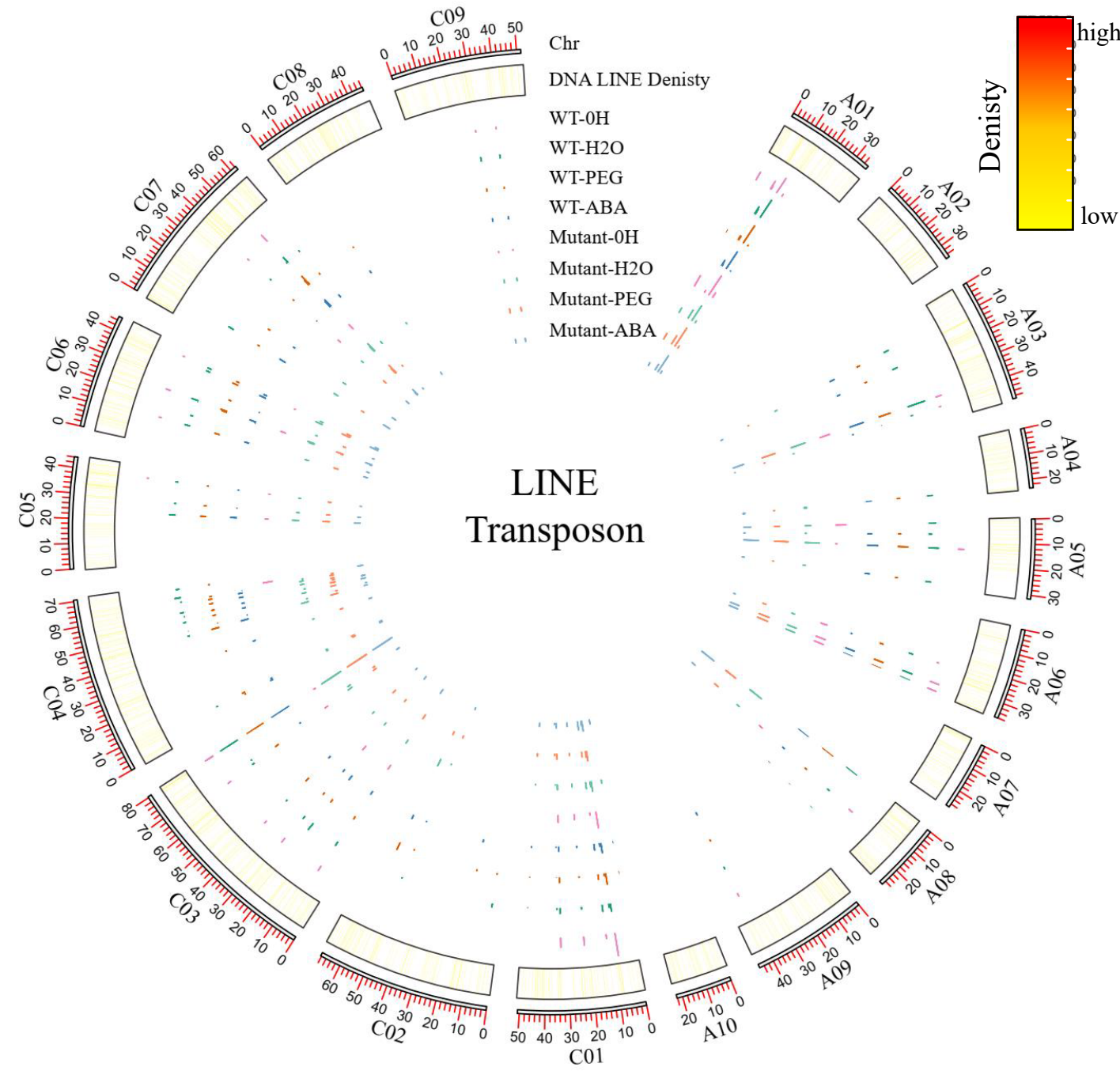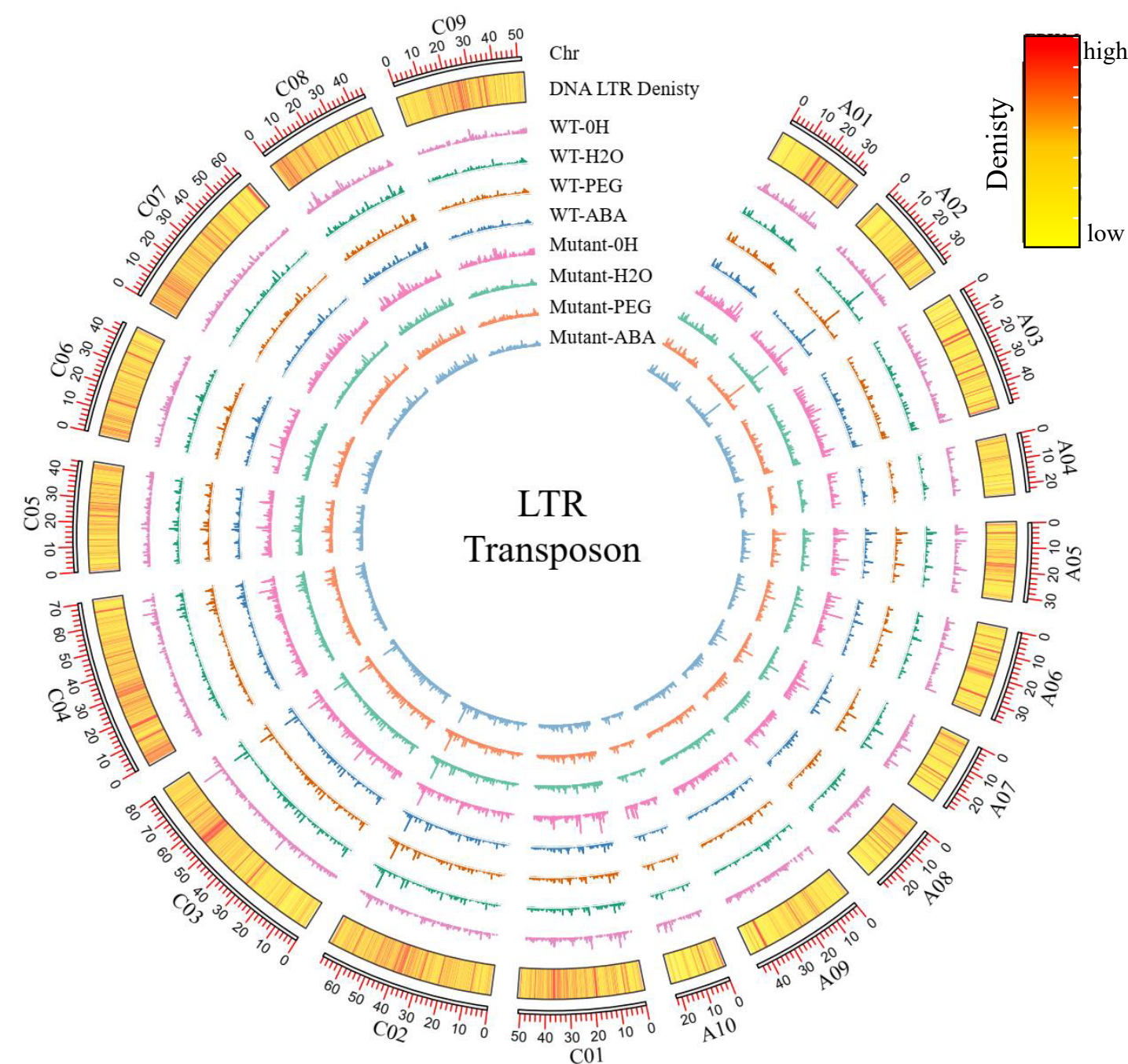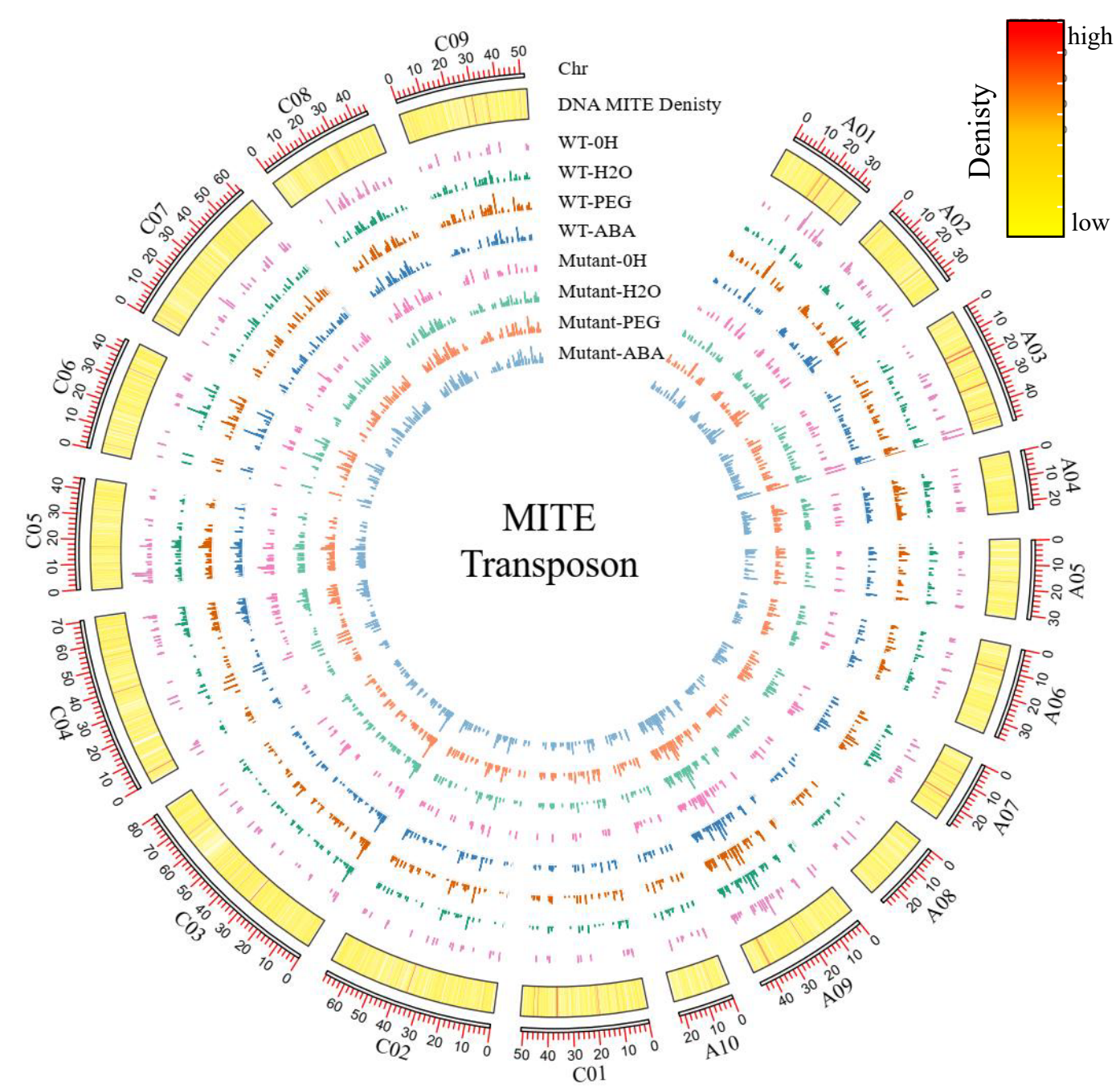

Supplement: Supplementary Figure 1 — Density distribution of LINE, LTR and MITE transposons across chromosomes and their expression patterns in various samples. The circos plot from outer to inner rings shows chromosomes, transposon density within 50 kb windows, and expression levels under different treatments across different samples within 50 kb windows. [file DataSheet1.pdf]

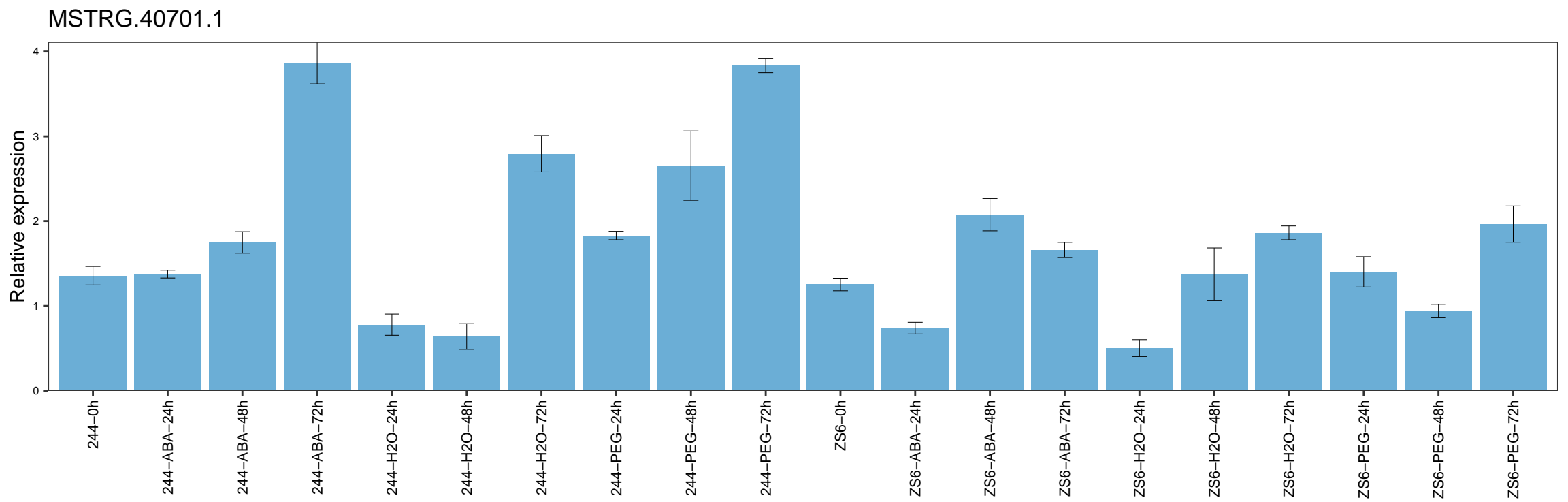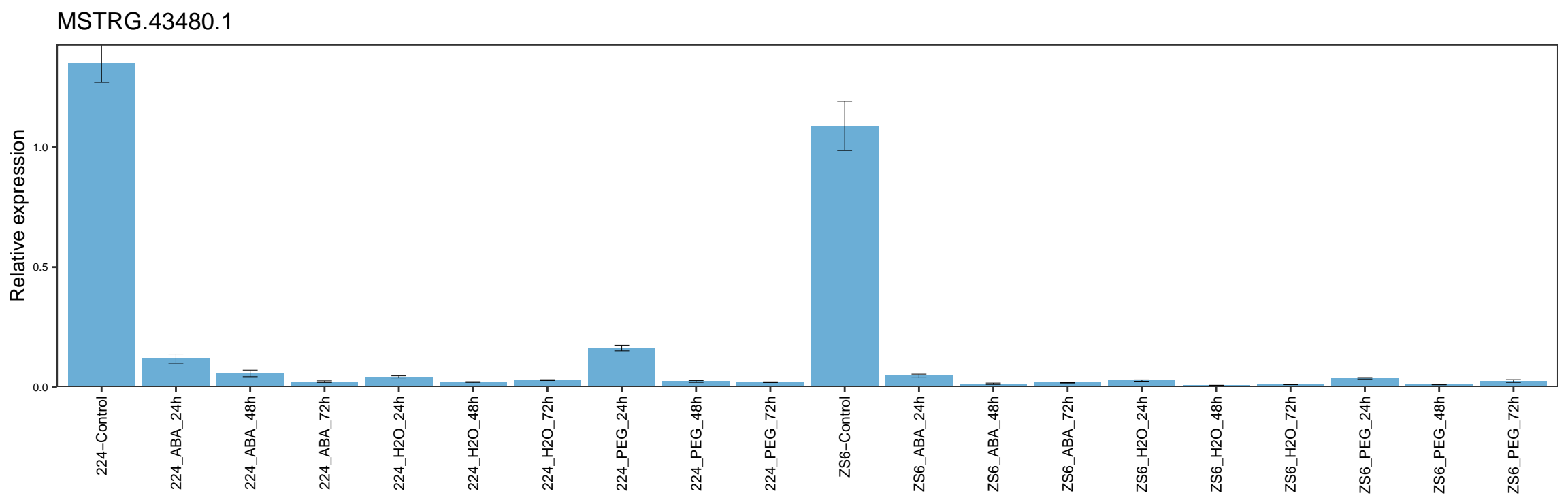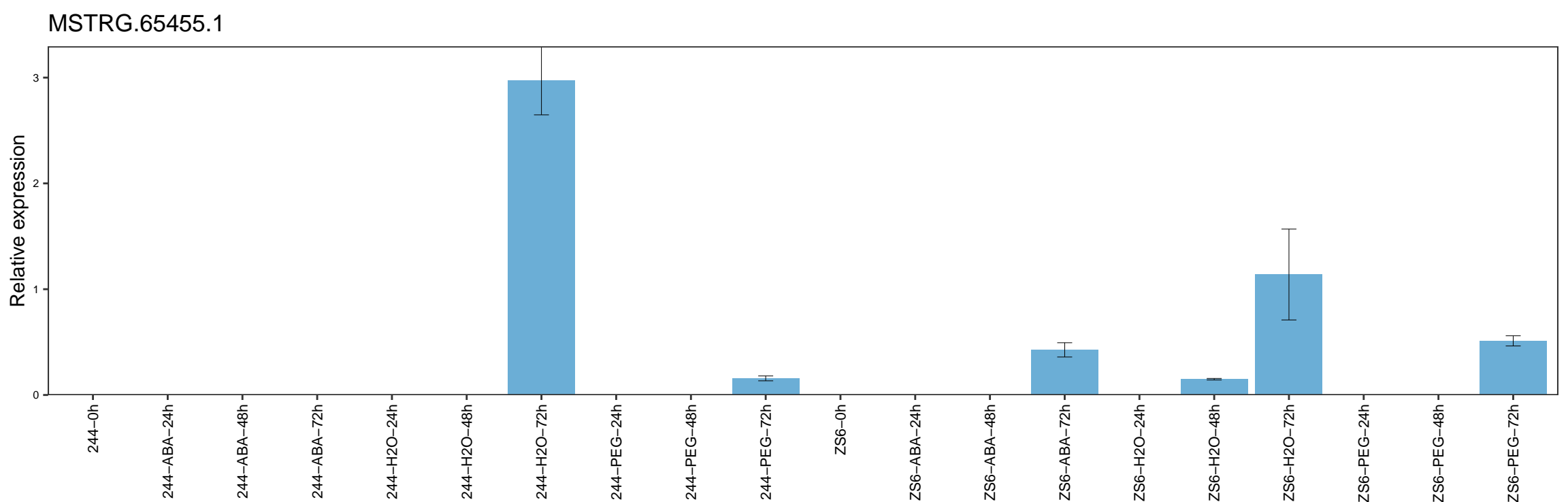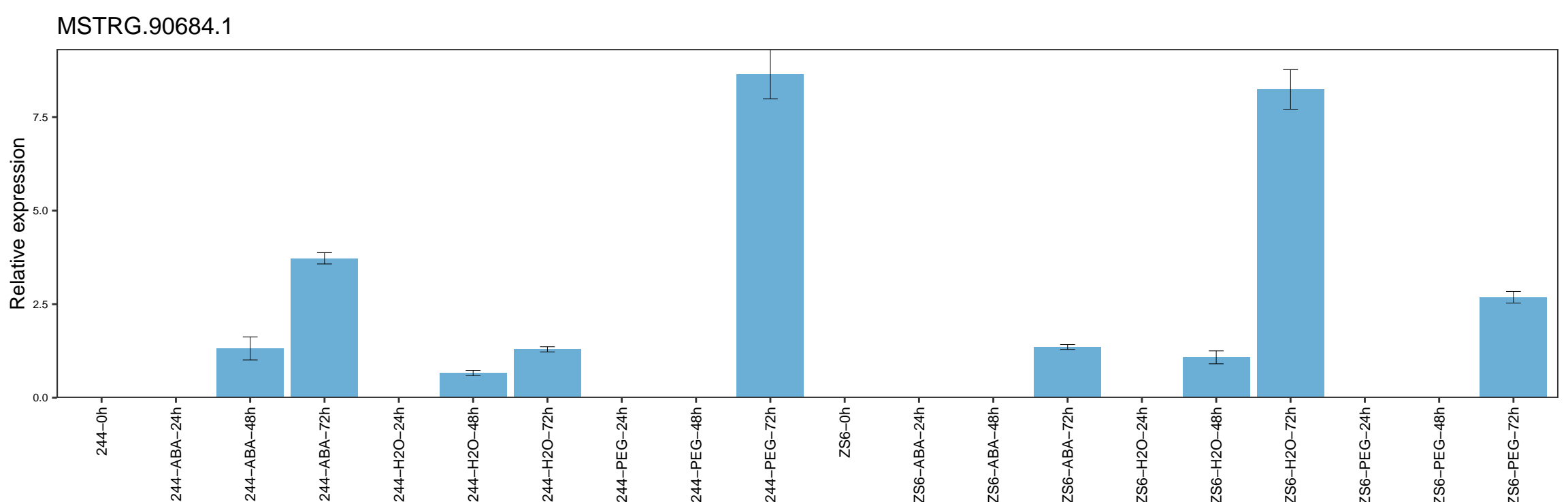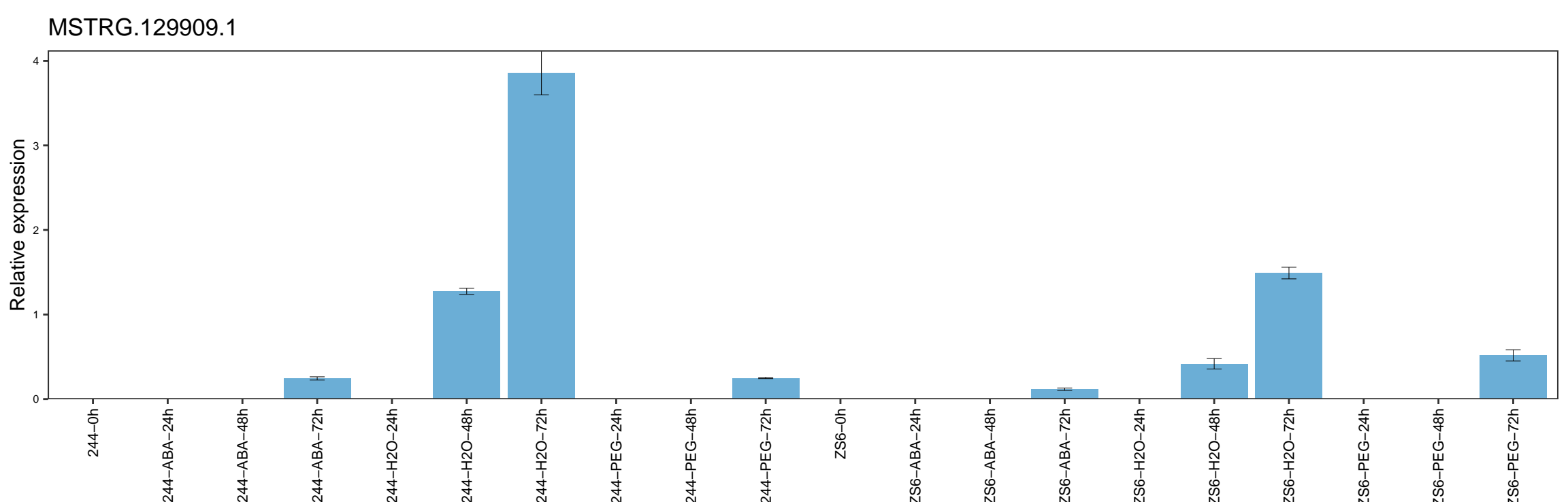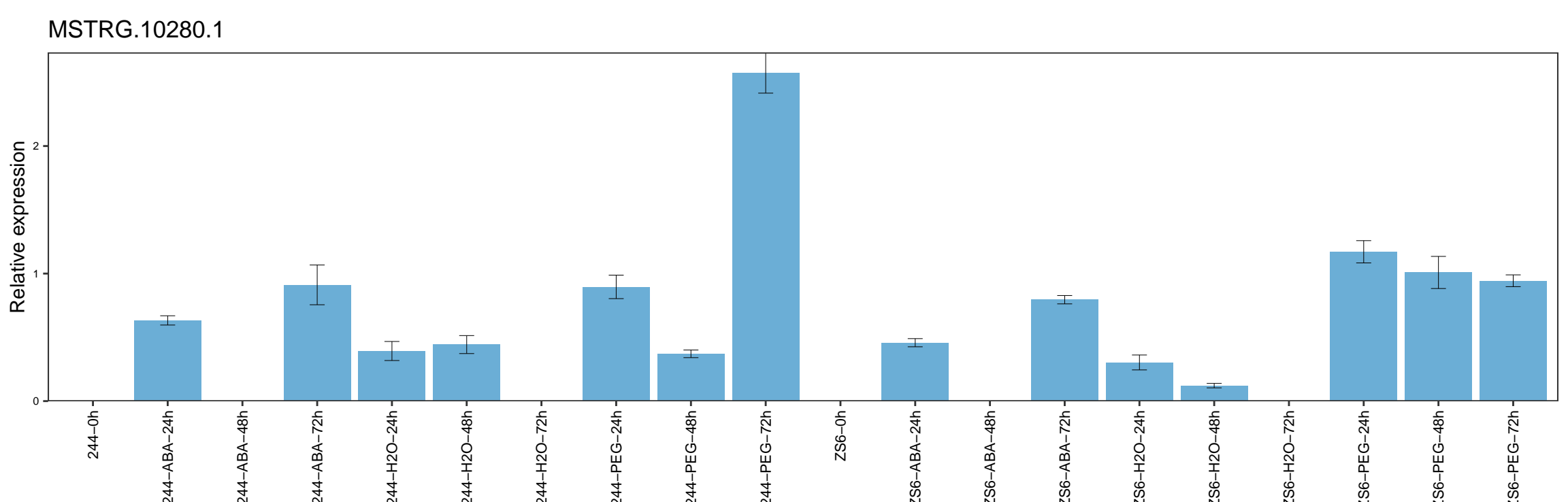

Supplement: Supplementary Figure 2 — The bar chart shows the relative expression levels quantified by qRT-PCR of six TE transcripts. The error line represents the standard deviation of three repetitions. [file DataSheet2.pdf]
